# Predicting prostate cancer in men with PSA levels of 4–10 ng/mL: MRI-based radiomics can help junior radiologists improve the diagnostic performance

**DOI:** 10.1038/s41598-023-31869-1

**Published:** 2023-03-24

**Authors:** Jian-Guo Zhong, Lin Shi, Jing Liu, Fang Cao, Yan-Qing Ma, Yang Zhang

**Affiliations:** Cancer Center, Department of Radiology, Zhejiang Provincial People’s Hospital, Affiliated People’s Hospital, Hangzhou Medical College, Hangzhou, Zhejiang China

**Keywords:** Cancer imaging, Prostate cancer

## Abstract

To develop MRI-based radiomics model for predicting prostate cancer (PCa) in men with prostate-specific antigen (PSA) levels of 4–10 ng/mL, to compare the performance of radiomics model and PI-RADS v2.1, and to further verify the predictive ability of radiomics model for lesions with different PI-RADS v2.1 score. 171 patients with PSA levels of 4–10 ng/mL were divided into training (n = 119) and testing (n = 52) groups. PI-RADS v2.1 score was assessed by two radiologists. All volumes of interest were segmented on T_2_-weighted imaging, diffusion weighted imaging, and apparent diffusion coefficient sequences, from which quantitative radiomics features were extracted. Multivariate logistic regression analysis was performed to establish radiomics model for predicting PCa. The diagnostic performance was assessed using receiver operating characteristic curve analysis. The radiomics model exhibited the best performance in predicting PCa, which was better than the performance of PI-RADS v2.1 scoring by the junior radiologist in the training group [area under the curve (AUC): 0.932 vs 0.803], testing group (AUC: 0.922 vs 0.797), and the entire cohort (AUC: 0.927 vs 0.801) (P < 0.05). The radiomics model performed well for lesions with PI-RADS v2.1 score of 3 (AUC = 0.854, sensitivity = 84.62%, specificity = 84.34%) and PI-RADS v2.1 score of 4–5 (AUC = 0.967, sensitivity = 98.11%, specificity = 86.36%) assigned by junior radiologist. The radiomics model quantitatively outperformed PI-RADS v2.1 for noninvasive prediction of PCa in men with PSA levels of 4–10 ng/mL. The model can help improve the diagnostic performance of junior radiologists and facilitate better decision-making by urologists for management of lesions with different PI-RADS v2.1 score.

## Introduction

Prostate cancer (PCa), with an estimated incidence of 7.1% and mortality rate of 3.8%, is the most common malignancy and the second leading cause of cancer-related death among men in the world^1^. Serum prostate-specific antigen (PSA) is widely used as a highly-sensitive screening tool for PCa. Serum PSA level of 4 ng/mL was previously considered as the cut-off point for performing biopsy^2, 3^. However, PSA level of 4–10 ng/mL is termed as the gray zone, as there is no clear consensus on the indication for performing biopsy in these patients^4, 5^. In previous studies, biopsy-positive rate in men with PSA levels of 4–10 ng/mL was less than 30%, which implies that up to 70% of men were overtreated^6, 7^. Unnecessary biopsy is a wastage of resources and can also lead to complications. Therefore, development of a non-invasive tool to predict PCa in patients who have PSA levels in the gray zone is a key imperative to reduce unnecessary biopsies.

The Prostate Imaging-Reporting and Data System was released in 2012 and updated in 2019 (PI-RADS v2.1)^8^. Currently, patients with PI-RADS score ≥ 3 are considered for prostate biopsy in clinical practice^9^. Of note, lesions with PI-RADS score of 3 are usually termed as "indeterminate" or "equivocal"^8^. Previous study has shown a low specificity of use of PI-RADS score of 3 as the cut-off point for PCa (62.1%), leading to a high proportion of false-positives^10^. Moreover, the PI-RADS score can be affected by the radiologists' subjectivity, and the most controversial lesions in this respect are those with PI-RADS score of 3. These limitations together with the development of increasingly powerful image acquisition and processing technologies have led to an increasing interest in new quantitative analysis methods.

Radiomics provides a non-invasive and low-cost automated technology for objective and quantitative evaluation of tumor heterogeneity^11, 12^. However, in the past five years, studies^13–16^ have mainly focused on PI-RADS score, patient’s age, prostate volume, PSA level, and other clinical indicators to predict the risk of PCa in men with PSA levels in the gray zone, while radiomics has rarely been applied in this field. Thus, there is still room for improvement in non-invasive tools to identify patients who are most likely to have PCa and select these patients for biopsy.

Therefore, the aim of this study was to develop MRI-based radiomics model as a non-invasive tool for predicting PCa in men with PSA levels of 4–10 ng/mL. In addition, we sought to compare the performance of radiomics model and PI-RADS v2.1, and to further verify the predictive ability of the radiomics model for lesions with different PI-RADS v2.1 score.

## Results

### Patient characteristics

A total of 171 patients with PSA in the gray zone were included in this study, including 66 patients with PCa and 105 patients with Non-PCa. In the evaluation of lesion location, 64 cases were located in the PZ and 107 cases were located in the TZ. Among the 66 PCa cases, 61 were clinically significant and 5 were clinically insignificant. Among the 105 patients with Non-PCa, there were 58 cases of benign prostatic hyperplasia and 47 cases of prostatitis. The characteristics of the study population are summarized in Table 1. The 171 included patients were divided into the training group (n = 119) and the testing group (n = 52). There were no significant between-group differences with respect to age, PSA level, lesion location, and PI-RADS v2.1 score (P = 0.435–0.973).

**Table 1:** Patients' characteristics in the training and testing groups.

Characteristics	Training group (n = 119)			Testing group (n = 52)			
	PCa (n = 43)	Non-PCa (n = 76)	*P*_*intra*_	PCa (n = 23)	Non-PCa (n = 29)	*P*_*intra*_	*P*_*inter*_
Age (years)	70.19 ± 8.38	66.33 ± 8.51	0.019	69.78 ± 8.02	66.17 ± 6.47	0.078	0.973
PSA (ng/mL)	7.69 ± 1.57	7.13 ± 1.59	0.068	7.41 ± 1.50	6.92 ± 1.71	0.279	0.464
Location			0.000			0.003	0.616
PZ	27	19		13	5		
TZ	16	57		10	24		
PI-RADS v2.1 score by Radiologist A			0.000			0.000	0.798
3	8	60		5	23		
4	28	13		13	5		
5	7	3		5	1		
PI-RADS v2.1 score by Radiologist B			0.000			0.000	0.435
3	6	72		6	27		
4	27	4		15	2		
5	10	0		2	0		

Note. PCa, prostate cancer; Non-PCa, Non-prostate cancer; PSA, prostate serum antigen; PZ, peripheral zone; TZ, transition zone. The assessment of PI-RADS v2.1 was performed by two radiologists, a junior with 5 years of experience (Radiologist A) and a senior with more than 20 years of experience (Radiologist B) in urogenital imaging. *P*_*intra*_ is the result of univariate analysis between PCa and Non-PCa while *P*_*inter*_ represents whether a significant difference exists between the training and testing groups.

On univariate analysis, significant differences were observed between PCa and Non-PCa patients in the training group with respect to age (P = 0.019), lesion location (P < 0.001), PI-RADS v2.1 score by Radiologist A (P < 0.001), and PI-RADS v2.1 score by Radiologist B (P < 0.001). In addition, there were significant differences with respect to lesion location (P = 0.003), PI-RADS v2.1 score by Radiologist A (P < 0.001) and PI-RADS v2.1 score by Radiologist B (P < 0.001) in the testing group.

### Performance of PI-RADS v2.1 between Radiologist A and Radiologist B

The weighted Cohen's kappa value was 0.584 (95% CI: 0.472–0.695, P < 0.001), indicating moderate overall inter-reader agreement with respect to PI-RADS v2.1 score. Among the 171 patients, 131 cases were consistently scored while 40 cases were inconsistently scored; of these 40 cases, 27 cases were over-graded by the Radiologist A. The PI-RADS v2.1 score of junior Radiologist A was more likely to be overestimated than that of Radiologist B (P = 0.030). Details of the PI-RADS v2.1 score between Radiologist A and Radiologist B are shown in the Table 2.

**Table 2:** The PI-RADS v2.1 score between Radiologist A and Radiologist B.

Radiologist B	Radiologist A			
	3	4	5	Total
3	88	18	5	111
4	8	36	4	48
5	0	5	7	12
Total	96	59	16	171
kappa value	0.584 (95%CI: 0.472–0.695, P < 0.001)			
McNemar test	P = 0.030			

Note. The assessment of PI-RADS v2.1 was performed by two radiologists, a junior with 5 years of experience (Radiologist A) and a senior with more than 20 years of experience (Radiologist B) in urogenital imaging.

### Comparative performance of radiomics model and PI-RADS v2.1

**Figure 1:**
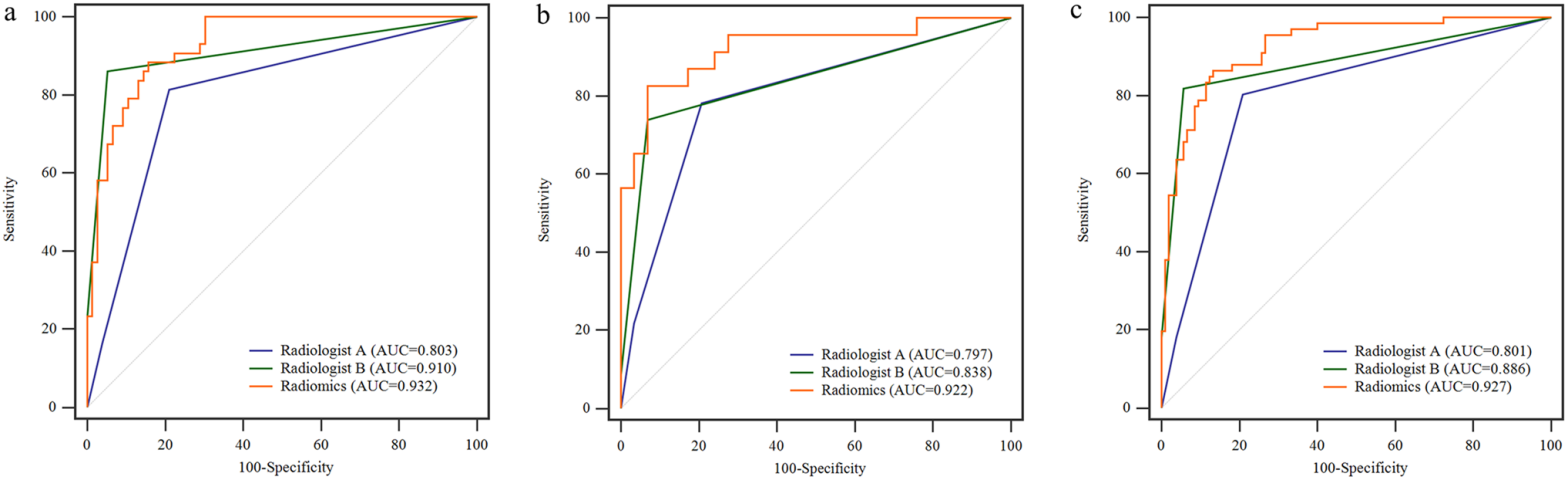
Receiver operating characteristic curves for prostate cancer prediction of radiomics model and PI-RADS v2.1 score by two radiologists (Radiologist A and Radiologist B) in the training (**a**), the testing (**b**), and the whole groups (**c**). Radiologist A, PI-RADS v2.1 score by a junior radiologist; Radiologist B, PI-RADS v2.1 score by a senior radiologist.

The diagnostic performance of radiomics model and PI-RADS v2.1 scoring by the two radiologists are shown in Table 3 and Fig. 1. The diagnostic performance of PI-RADS v2.1 score assigned by senior Radiologist B was better than that of junior Radiologist A in the training group (AUC: 0.910 vs 0.803, P = 0.0102), testing group (AUC: 0.838 vs 0.797, P = 0.4568), and the overall cohort (AUC: 0.886 vs 0.801, P = 0.0098). The radiomics model exhibited the best performance (AUC: 0.932, 0.922, and 0.927 in the training group, testing group, and the whole cohort, respectively). DeLong test showed that the radiomics model performed better than the PI-RADS v2.1 scoring by Radiologist A in the training group (P = 0.0042), testing group (P = 0.0472), and the entire cohort (P = 0.0006); however, there was no significant difference between radiomics model and PI-RADS v2.1 score assigned by Radiologist B (P = 0.1233–0.5126).

**Table 3:** Diagnostic performances of radiomics model and PI-RADS v2.1 score by two radiologists.

Groups	Models	AUC (95%CI)	SEN (%)	SPE (%)	PPV (%)	NPV (%)
Training	Radiologist A	0.803 (0.720–0.870)	81.4	79.0	68.6	88.2
	Radiologist B	0.910 (0.844–0.955)	86.1	94.7	90.2	92.3
	Radiomics	0.932 (0.871–0.970)	88.4	84.2	76.0	92.8
Testing	Radiologist A	0.797 (0.662–0.896)	78.3	79.3	75.0	82.1
	Radiologist B	0.838 (0.710–0.926)	73.9	93.1	89.5	81.8
	Radiomics	0.922 (0.813–0.978)	82.6	93.1	90.5	87.1
Whole	Radiologist A	0.801 (0.733–0.858)	80.3	79.1	70.7	86.5
	Radiologist B	0.886 (0.828–0.929)	81.8	94.3	90.0	89.2
	Radiomics	0.927 (0.877–0.961)	86.4	86.7	80.3	91.0

Note. AUC, area under the curve; SEN, sensitivity; SPE, specificity; PPV, positive predictive value; NPV, negative predictive value; CI, confidence interval. The assessment of PI-RADS v2.1 was performed by two radiologists, a junior with 5 years of experience (Radiologist A) and a senior with more than 20 years of experience (Radiologist B) in urogenital imaging.

### Validation of radiomics model

**Figure 2:**
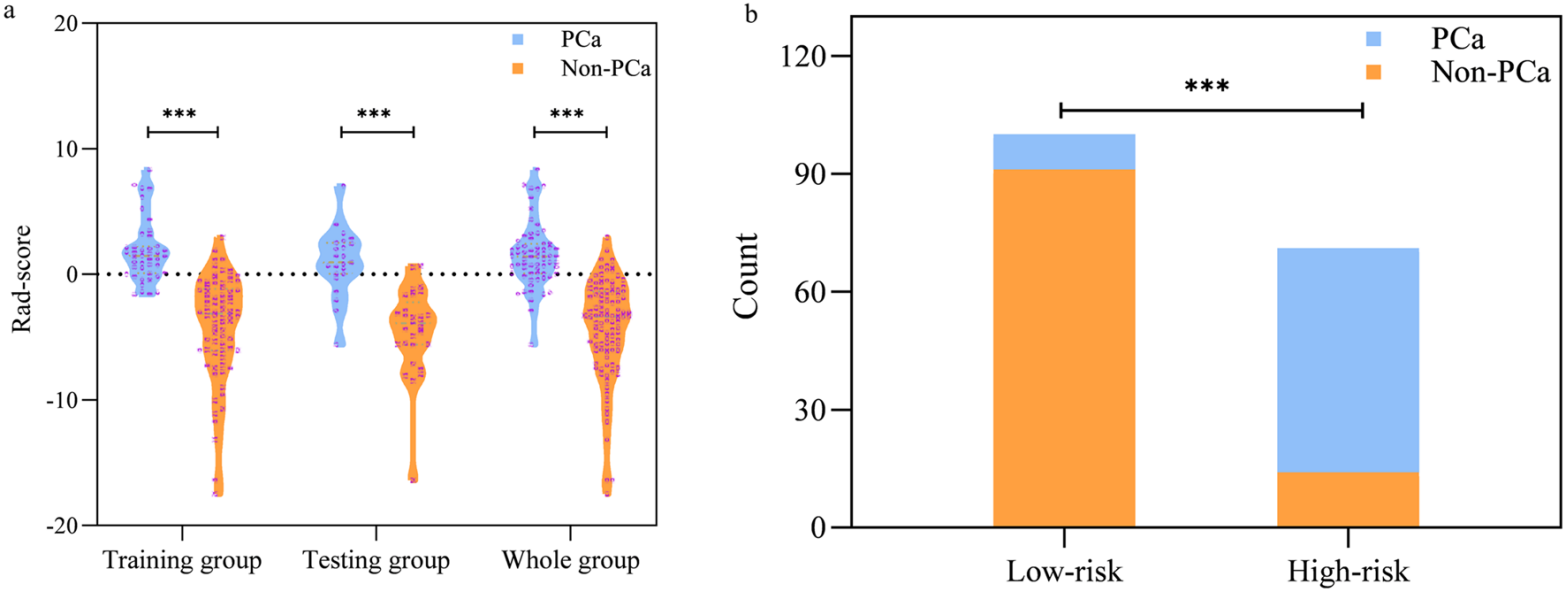
The rad-score violin plots of radiomics model (**a**). The probability of prostate cancer in the high-risk set was significantly higher than that in the low-risk set according to the radiomics model (**b**). *PCa* prostate cancer, *Non-PCa* non-prostate cancer; *** represents P < 0.001.

**Figure 3:**
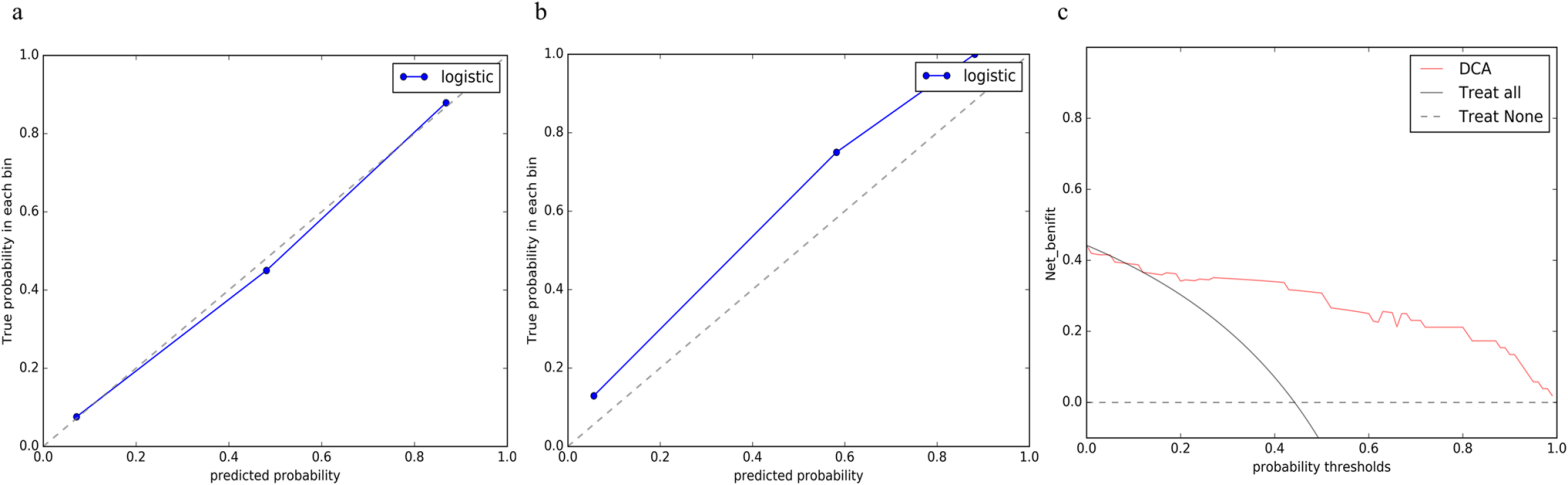
Calibration curves of radiomics model in the training (**a**) and the testing (**b**) groups. The decision curve analyses (DCA) of radiomics model in the testing group (**c**).

The rad-score of radiomics model showed significant differences between PCa and Non-PCa in the training group, testing group, and the entire cohort (P < 0.001, Fig. 2a). All patients were divided into high-risk set and low-risk set based on the cut-off value of -0.539. The probability of PCa in the high-risk set was significantly higher than that in the low-risk set according to the radiomics model (P < 0.001, Fig. 2b). The calibration curves demonstrated good agreement between the predicted and the actual probability of PCa (P = 0.484 and 0.139 in the training and testing groups, respectively; Hosmer–Lemeshow test) (Fig. 3a,b). DCA indicated that the net benefit for the radiomics model was higher than the measures that treat all patients and treat none patient in the testing group (Fig. 3c). The mean predicting accuracy of the five-fold cross-validation was 0.759.

**Figure 4:**
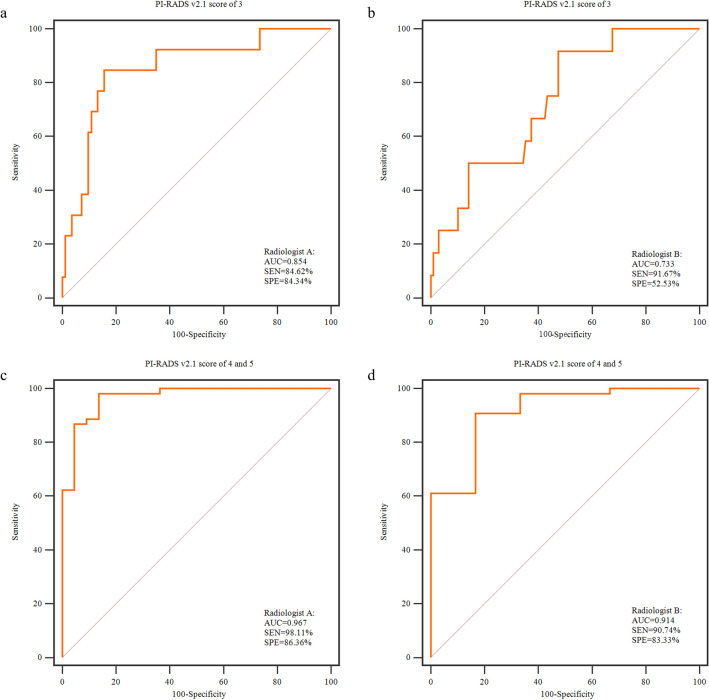
Verify the performance of radiomics model in lesions with PI-RADS v2.1 score of 3 (**a**,**b**) and PI-RADS v2.1 score of 4–5 (**c**,**d**) by junior Radiologist A (**a**,**c**) and senior Radiologist B (**b**,**d**) using receiver operating characteristic curves. Radiologist A, PI-RADS v2.1 score by a junior radiologist; Radiologist B, PI-RADS v2.1 score by a senior radiologist; *SEN* sensitivity, *SPE* specificity.

In addition, we further verify the performance of radiomics model for lesions with different PI-RADS v2.1 score. The radiomics model performed better for lesions with PI-RADS v2.1 score of 3 assigned by Radiologist A (AUC: 0.854, SEN: 84.62%, SPE: 84.34%) than those by Radiologist B (AUC: 0.733, SEN: 91.67%, SPE: 52.53%) for predicting PCa. And the radiomics model performed best for lesions with PI-RADS v2.1 score of 4–5 assigned by Radiologist A (AUC: 0.967, SEN: 98.11%, SPE: 86.36%) and Radiologist B (AUC: 0.914, SEN: 90.74%, SPE: 83.33%), as shown in Fig. 4.

## Discussion

In this study, we developed a MRI-based radiomics model to predict the risk of PCa in men with PSA levels of 4–10 ng/mL and compared its performance with the PI-RADS v2.1. Specifically, the radiomics model outperformed the results evaluated by the junior radiologist using PI-RADS v2.1 in the training group, testing group, and the entire cohort. A key finding was that the radiomics model also performed well for lesions with different PI-RADS v2.1 score.

In our study, we observed moderate inter-reader agreement of PI-RADS v2.1 (kappa value = 0.584), which was consistent with a previous study evaluating PI-RADS v2 scoring by experienced radiologists^17–19^. The advantage of our study was that we used the PI-RADS v2.1, which did not affect the overall diagnostic accuracy as compared to the PI-RADS v2, but helped improved the inter-reader consistency and simplified the allocation of scores^20, 21^. Another interesting finding was that the diagnostic performance of PI-RADS v2.1 score by senior radiologist was better than that by junior radiologist. Moreover, the junior radiologist tended to overestimate the PI-RADS v2.1 score (P = 0.030). This may be due to the complexity of prostate signal pattern and the fact that PI-RADS v2.1 mainly relies on subjective analysis of MRI ^22^. It must be acknowledged that the PI-RADS v2.1 score depends on the radiologist's experience and diagnostic capability^23^. Therefore, there is a need for development of more quantitative analysis methods.

Radiomics features offer the advantages of being an objective and quantitative tool. In our study, the radiomics model exhibited the best performance in both the training and testing groups, with an AUC of 0.932 and 0.922, respectively, which is consistent with the results of the two previous studies^24, 25^, with an AUC of 0.844 and 0.941, respectively. The other previous study^26^ employed radiomics features extracted from 2D segmentations to predict the risk of PCa in men with PSA levels of 4–10 ng/mL. However, compared with 2D segmentations, 3D segmentations can help reduce inter-reader variability; moreover, it better captures the heterogeneity of lesions, and helps reduce the sampling errors^27–30^. It is worth noting that our radiomics model performed better in the testing group (sensitivity: 82.6%, specificity: 93.1%, PPV: 90.5%, and NPV: 87.1%). This further demonstrated the ability of radiomics model for predicting PCa in men with PSA levels of 4–10 ng/mL, which can help decrease overdiagnosis and reduce unnecessary biopsies.

On comparing the ability of the radiomics model to predict PCa with that of PI-RADS v2.1, the radiomics model was found to perform slightly better than the PI-RADS v2.1 score by senior radiologist, but outperformed the PI-RADS v2.1 score by junior radiologist in the training, testing, and the entire cohort (P < 0.05). This finding was consistent with a previous study investigating patients with PSA at any level^31^. This is likely attributable to the fact that the PI-RADS v2.1 for predicting PCa is subjective and highly radiologist-dependent, whereas radiomics model is quantitative and relatively objective. In addition, we used a variety of methods to gradually filter the features to ensure that the selected features were valuable for predicting PCa. These encouraging results suggested that radiomics is a promising non-invasive technique for predicting PCa in men with PSA levels of 4–10 ng/mL. And in our study, we only included patients with PSA levels of 4–10 ng/mL to obtain a more precise diagnosis, thus resolving the clinical dilemma of whether biopsy should be performed.

Management decision-making for patients with lesions assigned PI-RADS v2.1 score of 3 is a clinical challenge for urologists: whether to follow up or conduct immediate biopsy^32^. The current guidelines do not clearly delineate the management strategy for these uncertain lesions^20, 33^. Previous report^34^ has investigated the usefulness of radiomics in detecting PCa in transition zone lesions with PI-RADS score of 3 (n = 41) and 4 (n = 32). However, the datasets were small and unbalanced, which limited the generalizability of the results. To the best of our knowledge, this is the first study that verified the predictive ability of the radiomics model for lesions associated with serum PSA levels of 4–10 ng/mL and PI-RADS v2.1 score of 3. Our study found that the radiomics model may potentially help predict PCa in lesions with PI-RADS v2.1 score of 3, thus facilitating the decision-making process for biopsy. Moreover, the radiomics model performed better for lesions with PI-RADS score of 3 assigned by junior radiologist (AUC: 0.854). This suggests that the radiomics model may be more useful for inexperienced radiologists in the PI-RADS v2.1 evaluation.

Some limitations of our study should be acknowledged. First, this was a retrospective single-center study without external validation. In future studies, our model should be assessed and verified in more centers with larger samples. Second, manual segmentations of VOIs are inevitably subjective bias. The VOIs were manually delineated by two radiologists in our study, and robust features were retained for further analysis to minimize the bias. Further studies should adopt automatic or semi-automatic segmentation. Third, we did not explore the performance of radiomics model in different zones because of the limited samples. Therefore, further investigations are needed to expand the samples and verify the predictive performance for PCa in different zones. Fourth, the pathological results were confirmed by biopsy. Nevertheless, we conducted multidisciplinary discussions before and after the biopsy to improve the accuracy and credibility. Additionally, we performed systematic TRUS-guided prostate biopsies plus targeted biopsies and identified lesions by comparing specimens with MRI results to reduce the deviation.

## Conclusion

In this study, the radiomics model quantitatively outperformed PI-RADS v2.1 for noninvasive prediction of PCa in men with PSA levels of 4–10 ng/mL. The radiomics model can help improve the diagnostic performance of junior radiologists and facilitate better management decision-making by urologists for lesions with different PI-RADS v2.1 score.

## Methods

### Patients

This retrospective study was approved by our institutional ethics committee. The need for informed consent was waived by the Zhejiang Provincial People’s Hospital ethics committee.

**Figure 5:**
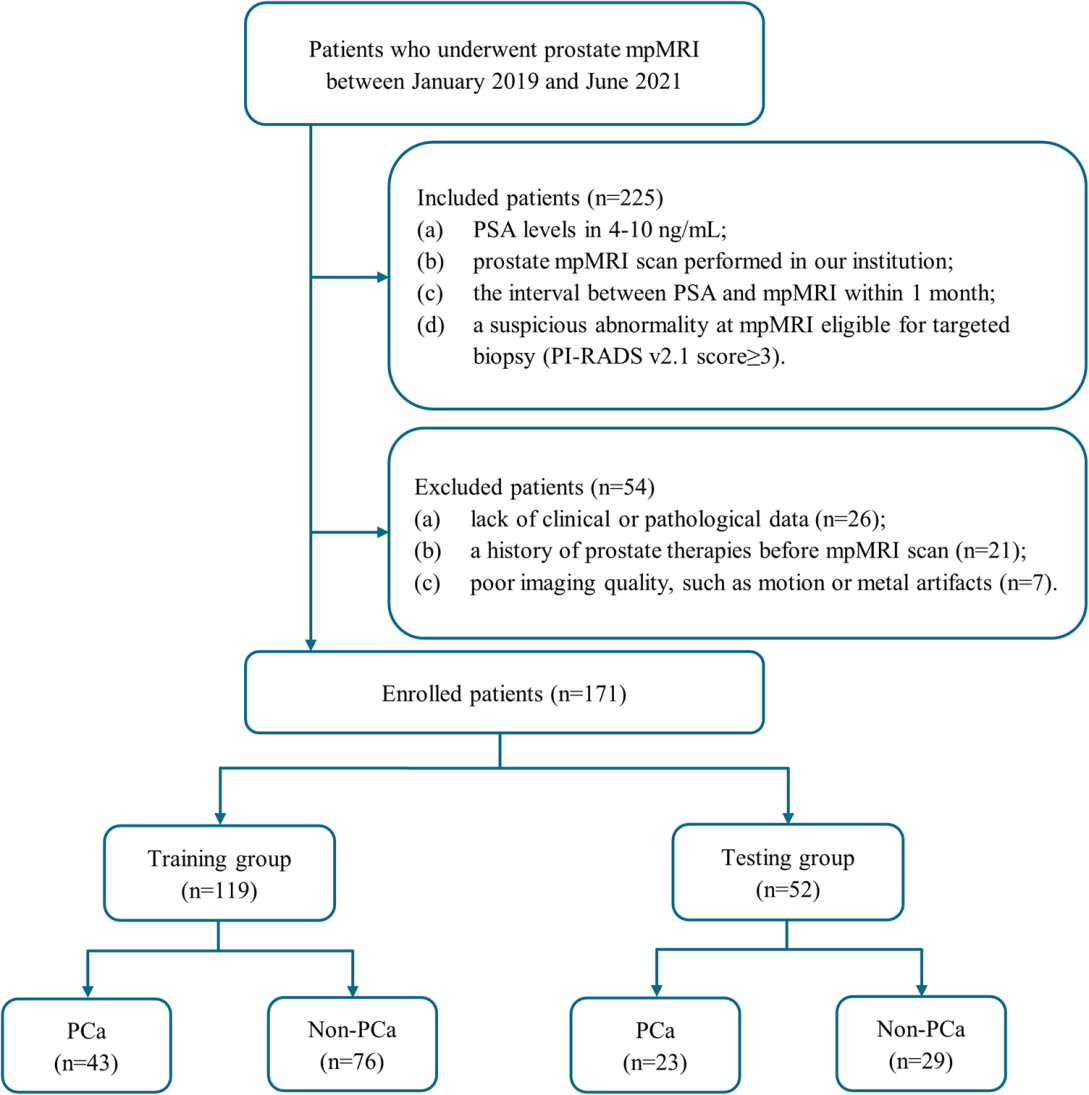
The study flowchart. *mpMRI* multiparametric magnetic resonance imaging, *PSA* prostate-specific antigen, *PCa* prostate cancer, *Non-PCa* non-prostate cancer.

Between January 2019 and June 2021, data pertaining to 225 patients were reviewed from the institutional picture archiving and communication system. The inclusion criteria were: (a) PSA levels 4–10 ng/mL; (b) prostate MRI scan performed at our institution; (c) ≤ 1 month interval between determination of PSA level and MRI; and (d) a suspicious abnormality in MRI eligible for targeted biopsy (PI-RADS v2.1 score ≥ 3). Out of 225 patients, 54 were excluded for the following reasons: (a) lack of clinical or pathological data (n = 26); (b) history of prostate therapies before MRI scan (n = 21); or (c) poor imaging quality, such as motion or metal artifacts (n = 7). Finally, 171 patients were enrolled and divided into training group (n = 119) and testing group (n = 52) at a ratio of 7:3 according to the time of MRI scan. The study flowchart is shown in Fig. 5.

The clinical data, including patient’s age, PSA level, lesion location, and PI-RADS v2.1 score were assessed.

### MRI scan

Prostate MRI with body phased-array coil was performed using a 3.0 T MRI scanner (Skyra, Siemens Healthcare, Erlangen, Germany), including axial T_1_-weighted imaging, axial and sagittal T_2_-weighted imaging (T_2_WI), axial diffusion weighted imaging (DWI), and dynamic contrast enhanced imaging (DCE). The temporal resolution for DCE imaging was 11 s, and the total scan time was 2 min. Apparent diffusion coefficient (ADC) sequence was automatically generated from the DWI sequence. The detailed parameters for each sequence are shown in Supplementary Materials I.

### PI-RADS v2.1 score

The PI-RADS v2.1 rates the likelihood of clinically significant cancer using a five-point scale: 1, very low; 2, low; 3, intermediate; 4, high; 5, very high. The assessment of PI-RADS v2.1 was performed by two radiologists: one junior radiologist with 5 years of experience (Radiologist A) and one senior radiologist with > 20 years of experience (Radiologist B) in urogenital imaging. Both radiologists were blinded to the initial MRI reports, clinical, and pathological data and respectively reviewed anonymized MRI images to assess the PI-RADS v2.1 score. For patients with multiple lesions, the largest or the most aggressive lesion in the PI-RADS v2.1 was assigned to the patient. Chi-squared test was used to analyze the PI-RADS v2.1 score assigned by the two radiologists, and weighted Cohen's kappa was used to assess inter-reader agreement.

### Pathological data

A multidisciplinary team comprising of radiologist, urologist, sonographer, and pathologist evaluated each case, before and after the biopsy, to guarantee that each patient's segmentation, PI-RADS v2.1 score, and biopsy results were matched to ensure accurate results. All patients underwent at least 12-core systematic transrectal ultrasound (TRUS)-guided prostate biopsy plus targeted biopsy of the suspicious lesion on MRI or prostatectomy within 3 months of MRI scan in order to obtain pathological results. At least two cores were obtained from targeted biopsy of the suspicious lesion. All biopsy or prostatectomy specimens were further processed and evaluated by two pathologists using the International Society of Urological Pathology-modified Gleason score classification^35^. Any differences in evaluation were resolved by consensus. The histopathological information, such as pathological type (e.g., benign prostatic hyperplasia, prostatitis, and PCa) and Gleason score (GS) were recorded. The pathological results were divided into two categories: PCa set (clinically significant PCa with GS ≥ 7 and clinically insignificant PCa with GS = 6) and Non-PCa set (benign prostatic hyperplasia and prostatitis).

### Image segmentation

Some preprocessing steps were carried out prior to feature extraction, namely resampling, normalization, and gray-level discretization using AK software (Artificial Intelligence Kit V3.0.0.R, GE Healthcare) to match the resolution^36–38^. Image preprocessing was performed by resampling the images with a resolution of 1 × 1 × 1 mm^3^ through the linear interpolation method. And we used a z-score normalization to make the image intensities fit a standard normal distribution with *μ* = 0 and *σ* = 1, where *μ* is the mean value, and *σ* is the variance. The desired minimum value of the initial setting of gray-level discretization is 0 and the desired maximum value is 255. Firstly, A.K. software was used to rigorously register the images of T_2_WI, DWI and ADC sequences in order to reduce the potential influence of the parameters of a scanning scheme. After that, the standardized T_2_WI images were imported into the ITK-SNAP software to manually segment the prostate index lesion of each patient layer by layer and to determine the three-dimensional (3D) volume of interest (VOI). Since the three sequences have been rigorously registered, tumor VOI obtained from T_2_WI can be applied directly to other sequences. Segmentations were performed by two radiologists (Radiologist A and Radiologist B). For patients with multiple tumor foci, only the index lesion with the highest PI-RADS v2.1 score or the largest volume was selected for analysis. All segmentations were performed completely blinded to the clinical and pathological data. Subsequently, Spearman rank correlation test was used to calculate the correlation coefficient (CC) for each feature between the feature sets from Radiologist A and Radiologist B, according to a previous study^39^. Features with CC values > 0.80 were considered as robust features^40^. The quantified values of robust features were averaged for further analysis.

### Radiomics analysis

A total of 1188 radiomics features were extracted for each patient using AK software. The details of all radiomics features are described in Supplementary Materials II. Firstly, 871 robust features were subjected using analysis of variance to preliminarily exclude the superfluous features, following which 559 radiomics features were retained. Then, minimum redundancy-maximum relevance algorithm was performed to eliminate the redundant and irrelevant radiomics features, following which 68 features were retained. Finally, the gradient boosting decision tree algorithm was used to reduce the dimension of the remaining features to select the most powerful radiomics feature subset, leading to retention of 14 features. Details of the remaining radiomics features are shown in Supplementary Materials III. We performed logistic regression analysis of these residual radiomics features to establish the radiomics model. Thereafter, we calculated the radiomics score (rad-score) for each patient. Details of the rad-score are shown in Supplementary Materials IV.

### Evaluation of PI-RADS v2.1 and Radiomics Model

**Figure 6:**
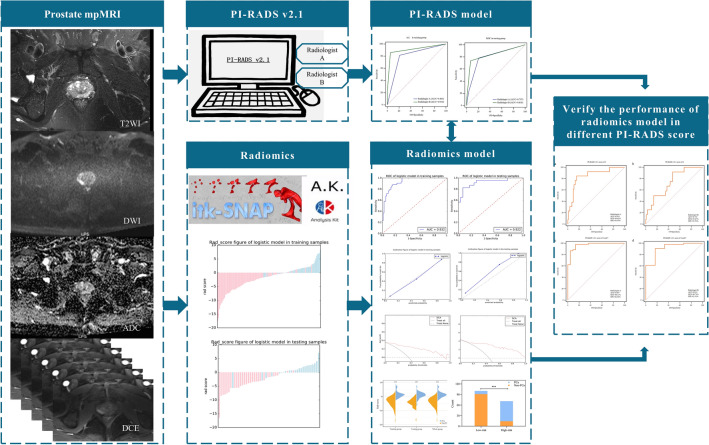
The radiomics workflow.

The diagnostic performance of PI-RADS v2.1 and radiomics model was evaluated using the receiver operative characteristic (ROC) curve analysis. The area under the curve (AUC), sensitivity, specificity, positive predictive value (PPV), and negative predictive value (NPV) were calculated in the training group, testing group, and the entire cohort, respectively. Respective AUC values were compared using the DeLong method to determine any significant difference. The agreement between radiomics model-predicted and expected probabilities was assessed using calibration curves and Hosmer–Lemeshow test. The clinical usefulness and the benefits of the radiomics model were evaluated using decision curve analysis (DCA). Five-fold cross-validation was also utilized for radiomics model evaluation. Finally, we used ROC curves to verify the predictive ability of radiomics model for lesions with different PI-RADS v2.1 score assigned by Radiologist A and Radiologist B, respectively. The radiomics workflow is shown in Fig. 6.

### Statistical analyses

Statistical analyses were performed using the SPSS software (version 24.0), MedCalc software (version 17.9.4), and R software (version 3.6.2). Normally distributed continuous variables with homogeneous variance are expressed as mean ± standard deviation and between-group differences assessed using independent sample *t* test. Non-normally distributed continuous variables are presented as median (interquartile range) and between-group differences assessed using Mann–Whitney U test. Categorical variables are presented as frequencies and between-group differences assessed using Pearson’s Chi-squared test or Fisher’s exact test. Two-sided P values < 0.05 were considered indicative of statistical significance.

### Ethics declarations

The study was approved by the institutional review board and the Zhejiang Provincial People’s Hospital ethics committee. This study was conducted in accordance with the relevant guidelines, national legislation and the institutional requirements. Written informed consent for participation was not required for this study in accordance with the national legislation and the institutional requirements.

## Supplementary Information

Supplementary Information.

## Data Availability

The datasets used and/or analyzed during the current study available from the corresponding author on reasonable request.
