# Supplementary material for: Predicting prostate cancer in men with PSA levels of 4–10 ng/mL: MRI-based radiomics can help junior radiologists improve the diagnostic performance
Source: Sci Rep. 2023 Mar 24;13:4846. doi: 10.1038/s41598-023-31869-1 (PMC10038986; doi:10.1038/s41598-023-31869-1)
Supplement: Supplementary file 1 — Supplementary Information. [file 41598_2023_31869_MOESM1_ESM.docx]

**Supplementary Materials:**

**Supplementary Materials I: MRI scan**

**Table S1.** The detailed parameters for each sequence.

| **Parameters** | **Sequences** | | | | |
| --- | --- | --- | --- | --- | --- |
|  | **T_1_WI** | **T_2_WI** | **T_2_WI** | **DWI** | **DCE** |
| Orientation | Axil | Axil | Sagittal | Axil | Axil |
| Technique | FSE | FSE | FR-FSE | SE-EPI | 3D-GRE |
| TR (ms) | 370 | 5300 | 4800 | 4900 | 5.0 |
| TE (ms) | 10 | 100 | 100 | 75 | 1.8 |
| Bandwidth (kHz) | 220 | 200 | 256 | 827 | 260 |
| Flip angle (degrees) | 126 | 160 | 120 | 180 | 12 |
| Echo train length | 3 | 25 | 25 | 120 | N/A |
| Matrix | 320×224 | 384×268 | 200×200 | 112×100 | 192×153 |
| FOV (mm^2^) | 200×200 | 200×200 | 320×224 | 200×180 | 200×200 |
| Thickness (mm) | 3 | 3 | 3 | 3 | 3 |

Note: TR, repetition time; TE, echo time; FOV, field of view; FSE, fast spin-echo; FR-FSE, fast-recovery fast spin-echo; SE-EPI, spin-echo echo-planar imaging; GRE, gradient echo.

**Supplementary Materials II: All radiomics features**

In this study, a total of 1188 radiomics features were extracted for each patient using AK software. The same 396 features were extracted from axial T_2_-weighted imaging (T_2_WI), diffusion weighted imaging (DWI), and apparent diffusion coefficient (ADC) imaging, respectively. These features could be divided into six groups: (I) Histogram (42 features), (II) gray-level cooccurrence matrix (GLCM, 144 features), (III) gray-level size zone matrix (GLSZM, 11 features), (IV) run-length matrix (RLM, 180 features), (V) Formfactor (9 features), and (VI) Haralick (10 features). These radiomics features were shown as follows.

**Supplementary Materials III: Remaining radiomics features**

We used Spearman rank correlation test, analysis of variance, minimum redundancy-maximum relevance algorithm and the gradient boosting decision tree algorithm to select the most optimal radiomics feature subset, and 14 features were retained. Details were as follows:

| **Sequences** | **Matrixes** | **Coefficient** | **Features** |
| --- | --- | --- | --- |
| T_2_WI | GLCM | -0.999 | GLCMEnergy_AllDirection_offset1_SD |
|  | GLCM | -0.595 | Correlation_AllDirection_offset7_SD |
|  | GLCM | 0.431 | ClusterShade_AllDirection_offset7_SD |
|  | GLSZM | 0.595 | SizeZoneVariability |
| DWI | GLCM | -1.804 | GLCMEnergy_AllDirection_offset1_SD |
|  | GLCM | 0.256 | ClusterProminence_angle135_offset4 |
|  | GLCM | 0.895 | ClusterProminence_AllDirection_offset1_SD |
|  | GLCM | 0.159 | HaralickCorrelation_AllDirection_offset4_SD |
|  | GLSZM | -1.335 | LowIntensityLargeAreaEmphasis |
|  | RLM | -0.349 | ShortRunEmphasis_angle135_offset7 |
| ADC | GLCM | 1.068 | ClusterProminence_AllDirection_offset1_SD |
|  | GLCM | 0.518 | ClusterShade_AllDirection_offset4 |
|  | RLM | 0.848 | ShortRunEmphasis_angle135_offset7 |
|  | RLM | 1.064 | LongRunLowGreyLevelEmphasis_AllDirection_offset4_SD |

Note: The value of intercept was -1.901. GLCM, gray-level cooccurrence matrix; GLSZM, gray-level size zone matrix; RLM, run-length matrix.

**Supplementary Materials IV: Radiomics score (rad-score)**

Calculation formula was as follows:

Rad-score = -1.901-0.999×T_2_WI_GLCMEnergy_AllDirection_offset1_SD

-0.595×T_2_WI_Correlation_AllDirection_offset7_SD

+0.431×T_2_WI_ClusterShade_AllDirection_offset7_SD

+0.595×T_2_WI_SizeZoneVariability

-1.804×DWI_GLCMEnergy_AllDirection_offset1_SD

+0.256×DWI_ClusterProminence_angle135_offset4

+0.895×DWI_ClusterProminence_AllDirection_offset1_SD

+0.159×DWI_HaralickCorrelation_AllDirection_offset4_SD

-1.335×DWI_LowIntensityLargeAreaEmphasis

-0.349×DWI_ShortRunEmphasis_angle135_offset7

+1.068×ADC_ClusterProminence_AllDirection_offset1_SD

+0.518×ADC_ClusterShade_AllDirection_offset4

+0.848×ADC_ShortRunEmphasis_angle135_offset7

+1.064×ADC_LongRunLowGreyLevelEmphasis_AllDirection_offset4_SD

The rad-score figures in training and testing samples were as follows. Blue represents prostate cancer (PCa) and red represents non-prostate cancer (Non-PCa). A score greater than 0 indicates PCa, and a score less than 0 indicates Non-PCa.


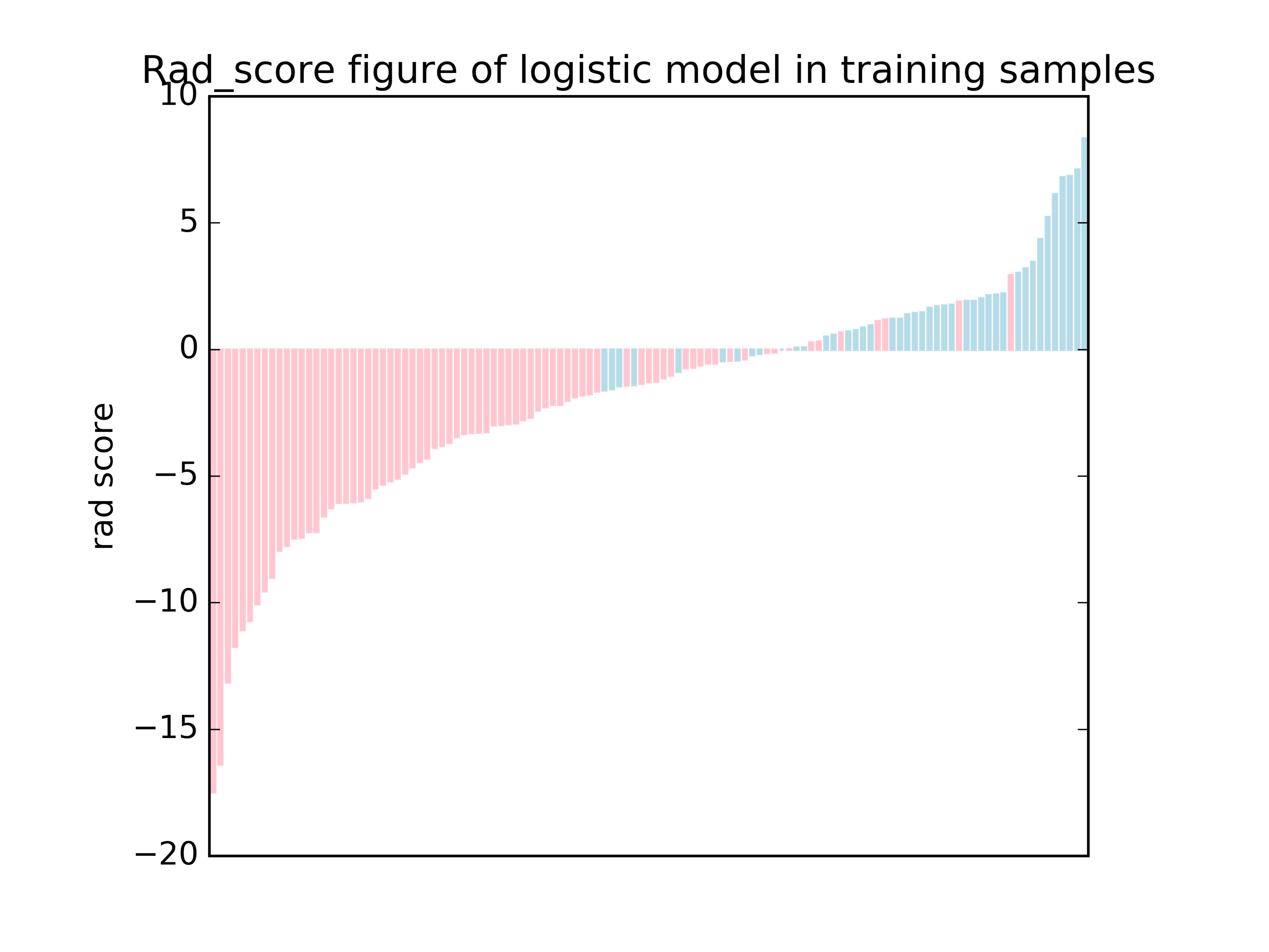
**
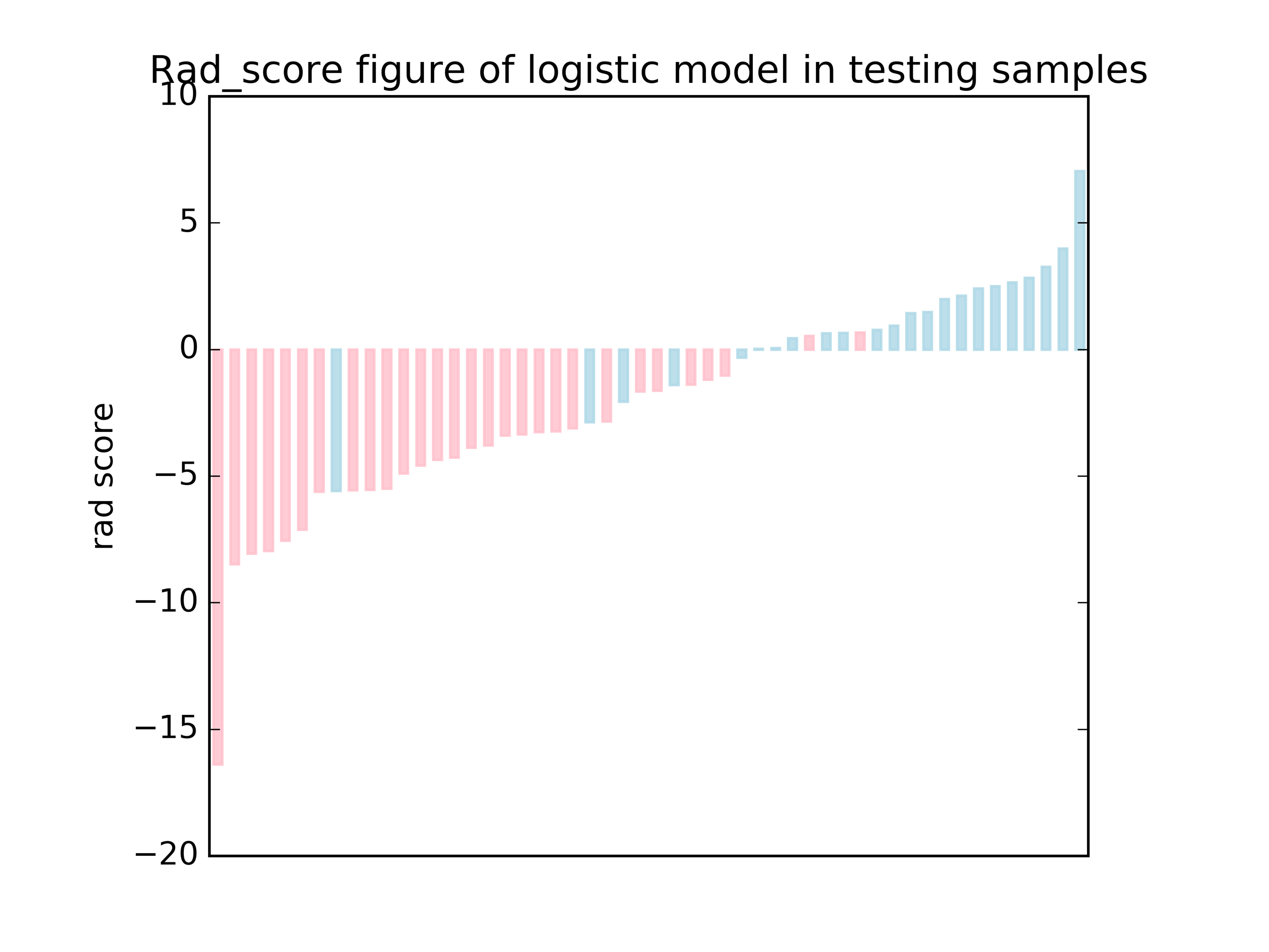
**
